# Monitoring of Total and Regional Liver Function after SIRT

**DOI:** 10.3389/fonc.2014.00152

**Published:** 2014-06-16

**Authors:** Roelof J. Bennink, Kasia P. Cieslak, Otto M. van Delden, Krijn P. van Lienden, Heinz-Josef Klümpen, Peter L. Jansen, Thomas M. van Gulik

**Affiliations:** ^1^Department of Nuclear Medicine, Academic Medical Center, Amsterdam, Netherlands; ^2^Department of Surgery, Academic Medical Center, Amsterdam, Netherlands; ^3^Department of Radiology, Academic Medical Center, Amsterdam, Netherlands; ^4^Department of Medical Oncology, Academic Medical Center, Amsterdam, Netherlands; ^5^Department of Hepatology, Academic Medical Center, Amsterdam, Netherlands

**Keywords:** radionuclide imaging, hepatobiliary imaging, SPECT/CT, hepatocellular carcinoma, liver function, selective internal radiation therapy

## Abstract

Selective internal radiation therapy (SIRT) is a promising treatment modality for advanced hepatocellular carcinoma or metastatic liver cancer. SIRT is usually well tolerated. However, in most patients, SIRT will result in a (temporary) decreased liver function. Occasionally patients develop radioembolization-induced liver disease (REILD). In case of a high tumor burden of the liver, it could be beneficial to perform SIRT in two sessions enabling the primary untreated liver segments to guarantee liver function until function in the treated segments has recovered or functional hypertrophy has occurred. Clinically used liver function tests provide evidence of only one of the many liver functions, though all of them lack the possibility of assessment of segmental (regional) liver function. Hepatobiliary scintigraphy (HBS) has been validated as a tool to assess total and regional liver function in liver surgery. It is also used to assess segmental liver function before and after portal vein embolization. HBS is considered as a valuable quantitative liver function test enabling assessment of segmental liver function recovery after regional intervention and determination of future remnant liver function. We present two cases in which HBS was used to monitor total and regional liver function in a patient after repeated whole liver SIRT complicated with REILD and a patient treated unilaterally without complications.

## Introduction

Radioembolization (also called selective internal radiation therapy or SIRT) is a form of brachytherapy in which intra-arterially injected ^90^ Y-loaded microspheres serve as sources for internal radiation purposes of non-resectable liver tumors. Average disease control ranges from 70 to 90% or even higher and SIRT is usually very well tolerated (1–3). Patients considered candidates for SIRT are patients with liver malignancies who do not qualify for surgical resection, radio frequent ablation, or transarterial chemoembolization. Most of these patients are classified as intermediate or advanced disease stage according to the Barcelona Clinic Liver Cancer scheme (4).

Severe side-effects of SIRT are not common after radioembolization. Several rather mild procedure-related symptoms may ensue including fatigue (54–61%), abdominal pain (23–56%), nausea and vomiting (20–32%), and low-grade fever (3–12%) that usually last only a few hours (5). Radioembolization appears to be well-tolerated and effective for the elderly as it is for younger patients with unresectable hepatocellular carcinoma (HCC) (6). Mild-to-moderate lymphopenia is commonly seen after radioembolization but not associated with increased susceptibility to infections (5). Main liver function related complications do not result from the microembolic effect of SIRT, even in patients with portal vein occlusion, but rather from an excessive irradiation of non-target liver tissue.

In a cirrhotic liver, the effect of radioembolization can affect the liver in two ways. On one hand, the usual distribution of microspheres can be profoundly altered by the vascular changes that occur in the cirrhotic liver. This altered microvascular pattern and the presence of anatomical arterio-portal and arterio-venous shunts may modify the radiation dose absorbed by the tumor and the non-tumoral liver and therefore affect treatment tolerance and effectiveness. On the other hand, the cirrhotic liver has a reduced functional reserve which increases the risk of liver failure after major liver surgery, liver insults including toxic or viral acute hepatitis and external irradiation. A direct liver cell injury and a further compromise of liver blood supply produced by radiation-mediated blood vessel damage could result in a higher risk of clinically relevant liver toxicity after radioembolization in comparison with non-cirrhotic livers (5).

## Radioembolization-Induced Liver Disease

Irradiation of the non-tumor liver tissue can result in an uncommon but potentially life-threatening condition after SIRT: radioembolization-induced liver disease (REILD). REILD has been recently distinguished from other liver toxicity diseases, such as radiation-induced liver disease (RILD) which is seen after external beam radiation, by the research group of Bilbao et al. (7). REILD is defined as a syndrome appearing 4–8 weeks after radioembolization as jaundice, mild ascites, and a moderate increase in GGT and alkaline phosphatize. It differs from RILD in the association with young age and the treatment intensity. It is seen in patients with liver cirrhosis or in patients who underwent chemotherapy prior or subsequent to SIRT (8). Recently, a modified treatment protocol with the aim to reduce the frequency and severity of REILD was presented by the same research group. In the modified protocol, the authors describe adjustments in the activity calculation together with the use of ursodeoxycholic acid and steroid therapy leading to a reduction in the frequency and severity of REILD. Although the study describes the largest cohort after SIRT so far, it lacks histopathological evidence. Without histopathological evidence, it is impossible to prove whether the clinical symptoms in cirrhotic patients are related to REILD or to progression of cirrhosis. Furthermore, the modified protocol implies lowering of the treatment activity. Though a decrease in treatment efficacy is not reported, we consider the evidence too little to introduce this new modified protocol in our daily practice.

## Liver Function

In clinical practice, we assume that SIRT will probably result in a (temporary) decreased liver function or occasional REILD. To minimize this risk, one can perform SIRT in two sessions enabling the primary untreated liver segments to guarantee liver function until function in the treated segments has recovered. At this moment, changes in the regional liver function after SIRT are unknown. The timing of the second SIRT-session is crucial as, if performed too early (i.e., before the function in the treated segments has recovered or the function in the non-treated lobes has compensated), it may cause the same complications as when performed in one session. In order to improve the current SIRT strategy, we need to investigate the changes in regional liver function after SIRT.

The liver is responsible for a spectrum of functions including the uptake, metabolism, conjugation, and excretion of various endogenous and foreign substances. Most of the different functions of the liver are performed by hepatocytes but to a smaller degree by other liver cells. The substrates, by-products, or final products of those processes can be used to estimate liver function. However, few of the tests used in everyday clinical practice truly measure liver function. They rather provide an indirect evidence of only one of the liver’s many processes.

Categorization schemes are based either on laboratory findings entirely or the combination of laboratory and clinical findings. The approach is to either stratify patients into groups with increasing degree of dysfunction or to calculate a score that reflects severity of liver dysfunction. The most widely used system today in adults is the model for end-stage liver disease (MELD) scoring system that uses the International Normalized Ratio (INR) in combination with serum bilirubin and serum creatinin values to predict survival (9). Another widely used scoring system is the Child–Pugh–Turcotte (CPT) score which includes total bilirubin serum level, albumin serum level, and prothrombin time together with the presence or absence of encephalopathy and ascites. The CPT scoring system categorizes patients into three groups (termed Child A, B, and C – in the order of increasing dysfunction) and is particularly useful in selecting patients with HCC and cirrhosis for resection or transplantation. Patients with liver metastases, who usually have normal liver parenchyma and who are classified as class Child A, are considered as candidates for liver resection. Detection of patients at risk for postoperative liver failure is of great importance. In these patients, the CPT score has been shown to be quite variable and may be unreliable for predicting the outcome of liver resections, especially in patients with non-cirrhotic livers (9).

The indocyanine green (ICG) clearance test was initially devised for the measurement of blood flow and later found clinical application in the assessment of liver function by yielding a measurement of functional hepatocyte mass. It is now the most widely used quantitative liver function test in clinical use in the setting of liver surgery. ICG is exclusively removed by the liver and excreted into the bile without intrahepatic conjugation. Elimination is dependent on hepatic blood flow, cellular uptake, and biliary excretion. The disappearance of ICG from the blood reflects the liver’s capacity to transport organic anions and metabolize drugs providing an indirect measurement of global liver function. A limitation is that ICG clearance test reflects global liver function, but it does not take into account regional variations in liver quality that may occur (10).

## Hepatobiliar Scintigraphy

Another frequently used quantitative liver function test is ^99m^Tc-mebrofenin hepatobiliary scintigraphy (HBS). ^99m^Tc-mebrofenin is a hepatic specific lidocaine analog and is transported to the liver bound to albumin and dissociates from albumin in the hepatic space of Disse from where it is taken up by the hepatocytes. Subsequently, mebrofenin is excreted into the bile canaliculi without prior biotransformation. The hepatic uptake represents one of the main hepatic processes and the use of ^99m^Tc-mebrofenin HBS for preoperative assessment of liver function in patients undergoing liver surgery was first described by Erdogan et al. In 54 patients scheduled for liver resection, the Mebrofenin uptake rate (MUR) strongly correlated with the ICG clearance test (11). HBS can be used in patients with normal and compromised liver parenchyma as the test is validated with a single cut-off value (2.69%/min/m^2^) for both categories of patients (12). Underlying liver disease is often unknown and remains unknown until liver resection is performed, if applicable. Liver biopsies are not taken routinely as the distribution of compromised parenchyma in the liver is not homogeneous leading to sampling errors and because of the risk of biopsy related complications. This is one of the main advantages of HBS compared to other modalities and increases its applicability in daily practice especially in patients with an uncertain quality of liver parenchyma.

Besides quantitative information, HBS provides visual information about the localization of liver segments with poor function. Because of the possibility of determining regional liver function, HBS was validated as a preoperative method for estimating future remnant liver (FRL) function. In this relatively small patient study, preoperatively estimated FRL function correlated well with actual remnant liver function 1 day after resection (10). A recent study including 36 patients demonstrated that ^99m^Tc-mebrofenin SPECT combined with subsequent low dose CT provided valuable visual information on the distribution of liver function (12). The results of functional liver volume measured by SPECT and morphologic volume measured by CT volumetry indicated that SPECT was an accurate method of measuring hepatic volume. FRL (corrected) cMUR measured by the combination of SPECT and dynamic HBS was able to accurately predict the actual function of the postoperative remnant liver. Preoperative portal vein embolization (PVE) is often performed in patients with insufficient FRL volume in order to achieve resectability. It induces atrophy of the embolized, tumor-bearing liver segments, while compensatory hypertrophy occurs in the non-embolized liver, thereby increasing FRL volume and function. PVE reduces the risk of postoperative liver insufficiency and enables resection of livers previously considered unresectable because of marginal FRL. Few studies have investigated the improvement in FRL function after PVE, since most quantitative liver function tests lack the ability to measure FRL function selectively. In a study by de Graaf et al., it was confirmed that the increase in FRL function was more pronounced than the increase in FRL volume after PVE (13). The uptake function in the FRL per liter of liver tissue increases after PVE. The increased liver function per gram or milliliter of liver tissue has been described previously during liver regeneration after partial hepatectomy. Combination of the ICG clearance test and the percentage of FRL volume derived by CT volumetry has been described for the assessment of FRL function after PVE. This method, however, is based on the assumption that the increase in FRL volume is related to the increase in FRL function, and that liver function is distributed homogeneously within the liver volume. However, only a weak correlation between the increase in FRL volume and FRL function was found, and so combination of the ICG clearance test with the percentage FRL volume derived from CT volumetry may not accurately measure FRL function after PVE. This indicates the importance of additional quantitative functional assays that specifically measure FRL function after PVE.

For liver surgery, HBS has been validated as a tool to assess total and regional liver function, and is performed routinely in our patients before major liver resection (14). It is also used to assess segmental liver function before and after PVE. HBS is considered as a valuable quantitative liver function test enabling assessment of segmental liver function recovery after regional intervention and determination of FRL function.

In this light, we consider ^99m^Tc-mebrofenin HBS a suitable quantitative liver function test to monitor regional liver function changes after SIRT. The effect of SIRT observed 4–6 weeks after the procedure is performed. HBS is performed before and 6 weeks after SIRT in order to measure the changes in the treated and non-treated lobes. If our hypothesis is correct and SIRT does cause decrease in liver function in the treated segments and (hyper)function in the untreated segments, HBS can be used as a monitoring tool in order to determine the timing of the second SIRT session.

## Case Examples

The two case examples presented here illustrate the possible role of total and regional functional imaging of the liver. Both patients were treated with SIR-Spheres for HCC in a salvage setting. Immediately before and 6 weeks after the SIRT, liver function was measured with HBS as described elsewhere (11).

It is shown that HBS is able to depict relative and absolute change in regional liver function in non-affected liver segments.

**Figure 1:**
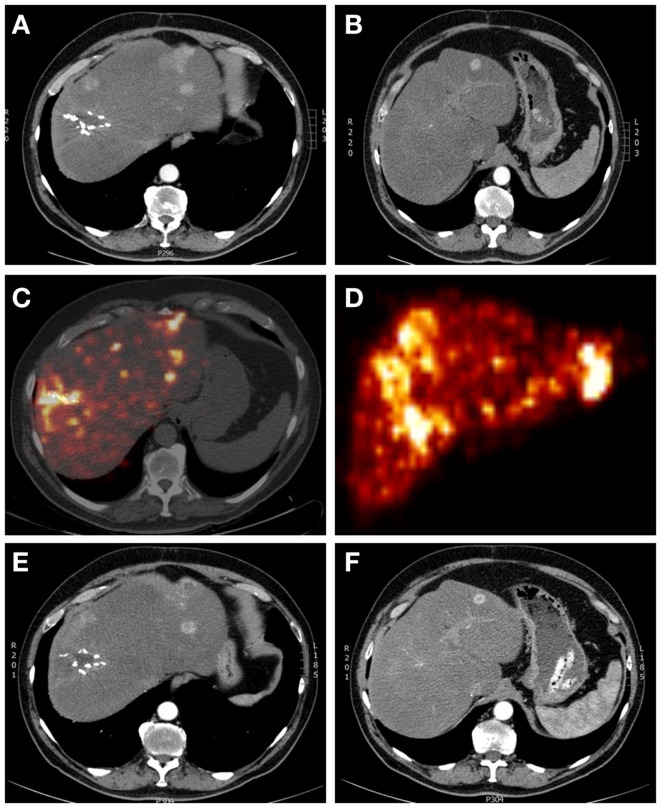
**A 65-year-old male patient with multifocal recurrent hepatocellular carcinoma (HCC) 6 months after resection of a HCC in liver segment 7–8**. **(A,B)** Arterial phase contrast-enhanced CT showing operation clips *in situ* after resection of the primary HCC and multiple sites of contrast enhancement (with wash-out on delayed images – not shown). **(C)** Yttrium-90 PET-lowdose CT and **(D)** maximum intensity projection after treatment with 1.7 GBq ^90^ Y labeled SIR-Spheres, showing markedly increased uptake of SIR-Spheres at the site of resection and multifocal in both right and left liver lobes, with clear right-sided predominancy. **(E,F)** Arterial phase contrast-enhanced CT showing response with central necrosis in tumor lesions, with no significant changes in liver volume.

**Figure 2:**
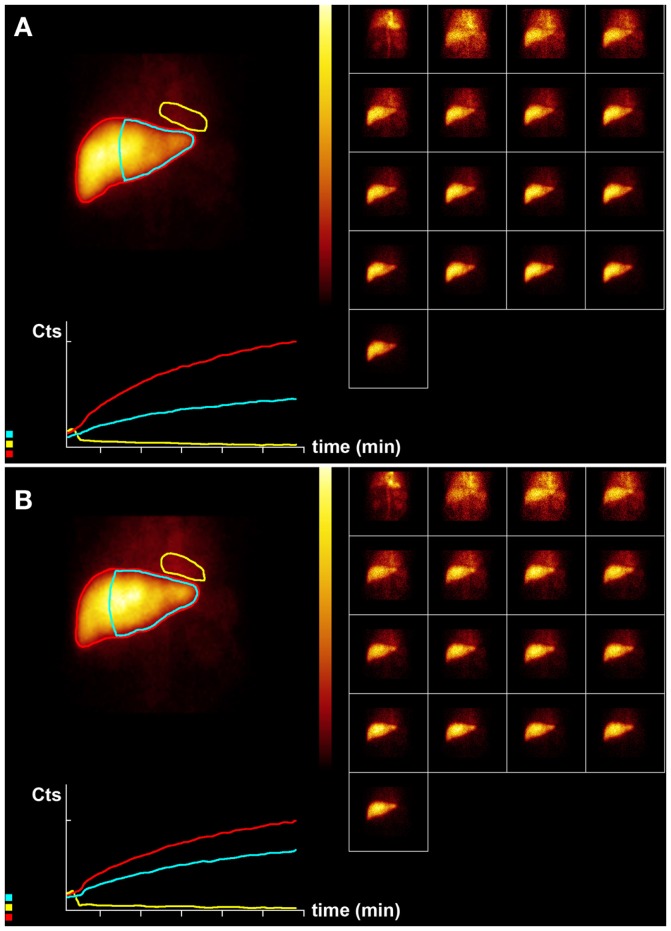
**Hepatobiliary scintigraphy before (A) and 6 weeks after (B) whole liver treatment with 1.7 GBq ^90^ Y labeled SIR-Spheres in a 65-year-old male patient with multifocal predominantly right-sided recurrent hepatocellular carcinoma (Figure 1)**. Displayed are summed dynamic frames (2 × 10 s/frame), a summed image (20 × 10 s) with regions of interest drawn around the entire liver (red), left liver segments 2–4 (blue), and cardiac blood pool (yellow), and corresponding time–activity curves. The total liver function (body surface area corrected mebrofenin uptake rate) was reduced after treatment from 7.4 to 6.1%/min (red curves). However, function of left liver lobes 2–4 increased from 3.4 to 4.1%/min (blue). This functional increase is both relative and absolute and can be explained by insufficient post treatment functional liver capacity leading to functional hypertrophy in non-affected liver segments.

**Figure 3:**
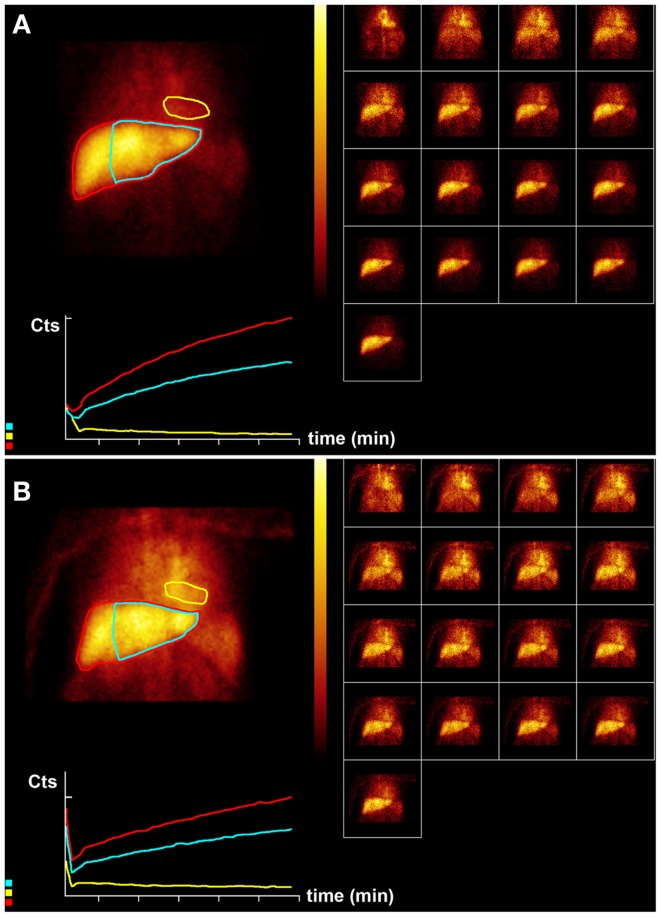
**Hepatobiliary scintigraphy before (A) and 6 weeks after (B) a second treatment (6-month interval) with 1.5 GBq ^90^ Y labeled SIR-Spheres in a 65-year-old male patient with multifocal predominantly right-sided recurrent hepatocellular carcinoma (Figure 1)**. Displayed are summed dynamic frames (2 × 10 s/frame), a summed image (20 × 10 s) with regions of interest drawn around the entire liver (red), left liver segments 2–4 (blue), and cardiac blood pool (yellow), and corresponding time–activity curves. The total liver function (body surface area corrected mebrofenin uptake rate) was reduced after treatment from 4.8 to 2.2%/min (red curves). This time, function of left liver lobes 2–4 decreased proportionally from 3.2 to 1.6%/min (blue). The patient was diagnosed with radiation embolization induced liver disease.

Case 1: Multifocal HCC, SIRT whole liver treatment twice (Figures 1–3).

**Figure 4:**
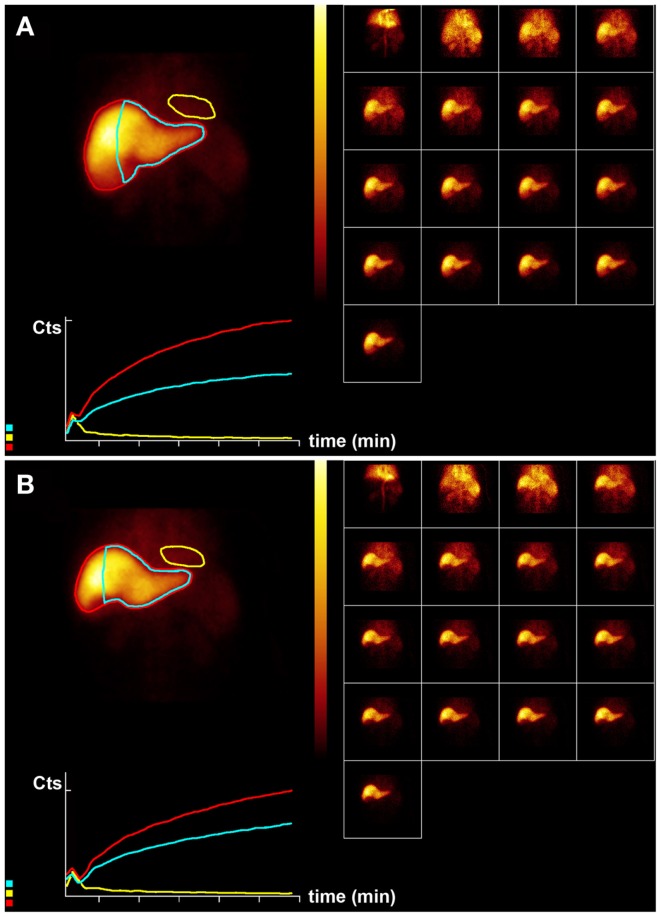
**Hepatobiliary scintigraphy before (A) and 6 weeks after (B) right-sided treatment with 1.8 GBq ^90^ Y labeled SIR-Spheres in a 64-year-old male patient with multifocal exclusively right-sided hepatocellular carcinoma with portal vein invasion**. Displayed are summed dynamic frames (2 × 10 s/frame), a summed image (20 × 10 s) with regions of interest drawn around the entire liver (red), left liver segments 2–4 (blue), and cardiac blood pool (yellow), and corresponding time–activity curves. The total liver function (body surface area corrected mebrofenin uptake rate) was reduced after treatment from 8.5 to 6.9%/min (red curves). However, function of left liver lobes 2–4 remained unchanged 4.8%/min (blue). This functional increase is only relative and can be explained by lack of functional hypertrophy in the presence of sufficient post treatment functional liver capacity.

Case 2: Multifocal HCC, SIRT right-sided liver segments (Figure 4).

## Concluding Remarks

Hepatobiliary scintigraphy is able to give insight into relative liver function before SIRT and depict relative and absolute change in regional liver function in non-affected liver segments after SIRT. This could be of importance in risk stratification of patients before the first treatment or timing of subsequent treatment.

## Author Contributions

Roelof J. Bennink, Kasia Cieslak, Heinz-Josef klümpen, and Thomas van Gulik were involved in study design, implementation, analysis, and manuscript preparation. Otto van Delden and Krijn van Lienden were involved in radioembolization, angiographic analysis, and manuscript preparation. Peter Jansen was involved in study design, clinical care, and manuscript preparation.

## Conflict of Interest Statement

The authors declare that the research was conducted in the absence of any commercial or financial relationships that could be construed as a potential conflict of interest.
